# Interactions of *Salmonella* with animals and plants

**DOI:** 10.3389/fmicb.2014.00791

**Published:** 2015-01-21

**Authors:** Agnès Wiedemann, Isabelle Virlogeux-Payant, Anne-Marie Chaussé, Adam Schikora, Philippe Velge

**Affiliations:** ^1^Institut National de la Recherche Agronomique, UMR1282 Infectiologie et Santé PubliqueNouzilly, France; ^2^UMR1282 Infectiologie et Santé Publique, Université François RabelaisTours, France; ^3^Institute for Phytopathology, Research Center for BioSystems, Land Use and Nutrition (IFZ), Justus Liebig University GiessenGiessen, Germany

**Keywords:** *Salmonella* infections, adhesion, invasion mechanisms, multiplication, host defense strategies

## Abstract

*Salmonella enterica* species are Gram-negative bacteria, which are responsible for a wide range of food- and water-borne diseases in both humans and animals, thereby posing a major threat to public health. Recently, there has been an increasing number of reports, linking *Salmonella* contaminated raw vegetables and fruits with food poisoning. Many studies have shown that an essential feature of the pathogenicity of *Salmonella* is its capacity to cross a number of barriers requiring invasion of a large variety of cells and that the extent of internalization may be influenced by numerous factors. However, it is poorly understood how *Salmonella* successfully infects hosts as diversified as animals or plants. The aim of this review is to describe the different stages required for *Salmonella* interaction with its hosts: (i) attachment to host surfaces; (ii) entry processes; (iii) multiplication; (iv) suppression of host defense mechanisms; and to point out similarities and differences between animal and plant infections.

## INTRODUCTION

The genus *Salmonella* consists of only two species, *S. bongori* and *S. enterica*, and the latter is divided into six subspecies: *enterica*, *salamae*, *arizonae*, *diarizonae*, *houtenae*, *indica. S*. *enterica* subsp. *enterica* includes more than 1,500 serotypes, which despite their high genetic similarity vary greatly in their host range and disease outcome ranging from enteritis to typhoid fever (Ohl and Miller, 2001). *Salmonella enterica* subsp. *enterica* is an important economic and public health problem throughout the world.

The degree of adaptation to hosts varies between *Salmonella* serotypes and determines the pathogenicity. Serotypes adapted to humans, such as *S*. Typhi and *S*. Paratyphi A, B, C, cause systemic typhoid fever. These serotypes are not pathogenic for animals. Similarly, *S*. Gallinarum and *S.* Abortusovis, which are specifically adapted to poultry and ovine, respectively, are responsible for severe systemic infections in these animals. However, *S*. Choleraesuis, for which pigs are the primary hosts, also causes severe systemic illness in humans. Ubiquitous serotypes, such as *S.* Enteritidis or *S.* Typhimurium, generally cause gastrointestinal infections in humans but can induce other diseases in animals (Hoelzer et al., 2011). For example, they can produce typhoid-like infections in mice, systemic infection in humans or asymptomatic intestinal colonization in chickens and pigs (Velge et al., 2012). Some of them are responsible for chlorosis on plant leaves sometimes causing death (Klerks et al., 2007b; Schikora et al., 2008, 2011; Gu et al., 2013b).

Disease in mammals occurs after ingestion of contaminated food or water. *Salmonella* infection of animals and humans depends on the ability of bacteria to survive the harsh conditions of the gastric tract before entering the intestinal epithelium and subsequently colonizing the mesenteric lymph nodes and internal organs in the case of systemic infections. In order to enter non-phagocytic cells and survive within the host environment, *Salmonella* has evolved mechanisms to interact with host cells and to induce its own internalization (Vazquez-Torres et al., 1999; Rosselin et al., 2012).

*Salmonella* usually enters agricultural environments via animal feces. Animals can directly contaminate plants or surface water used for irrigation and pesticide or fertilizer diluent through contaminated feces. Recently, there has been an increasing number of reports, linking *Salmonella* contaminated raw vegetables and fruits with food poisoning (Heaton and Jones, 2008). *Salmonella* is able to adapt to different external conditions including low pH or high temperature, allowing it to survive outside the host organism (Samelis et al., 2003; Semenov et al., 2007). Indeed, *Salmonella* is able to attach and adhere to plant surfaces before actively infecting the interior of different plants, leading to colonization of plant organs (Klerks et al., 2007a; Gu et al., 2011), and suppression of the plant immune system (Schikora et al., 2012). In addition, *Salmonella* originating from plants retains virulence toward animals (Schikora et al., 2011). Thus, plants are an alternative host for *Salmonella* pathogens, and have a role in its transmission back to animals.

Currently it is poorly understood how *Salmonella* successfully infects hosts as diversified as humans, animals, or plants. Here, our current understanding of the strategies used by *Salmonella* to colonize mammals and plants will be summarized. The gap in our knowledge about the differences in host colonization between animals and plants will be discussed.

## COLONIZATION

*Salmonella* infection requires different stages: attachment and adhesion to host surfaces, and production of bacterial factors, which facilitate invasion, initial multiplication, and ability to overcome or bypass host defense mechanisms.

### ADHESION TO HOST SURFACES

One of the first crucial events in successful colonization by *Salmonella* is adhesion to tissues. Two steps can be distinguished in the adhesion process: an initial adhesion that is reversible followed by a tight attachment which depends on bacterial factors and that is irreversible (Cevallos-Cevallos et al., 2012). This first contact is decisive whatever the host infected. However, this step is not exactly the same in animals and plants. In animals, bacterial adhesion occurs when *Salmonella* interacts with eukaryotic cells prior to invasion or when bacteria initiate biofilm formation on host surfaces, such as the intestinal epithelium or gallstones. In contrast, to date bacterial adhesion has been described only at the plant surface level and not at the plant cell level. Nevertheless, as in animals, biofilm formation on plant tissue has been observed to play an important role in plant colonization by *Salmonella* often in association with other plant pathogens as described in Section “The Different Multiplication Areas of *Salmonella*.” To strongly adhere to surfaces, *Salmonella* serotypes use several surface components depending on the surface to which they will attach. The different adhesive structures of *Salmonella*, their host receptor when known and the current knowledge about their role in the interaction of *Salmonella* with animals and plants are described below.

#### Fimbrial structures

**FIGURE 1:**
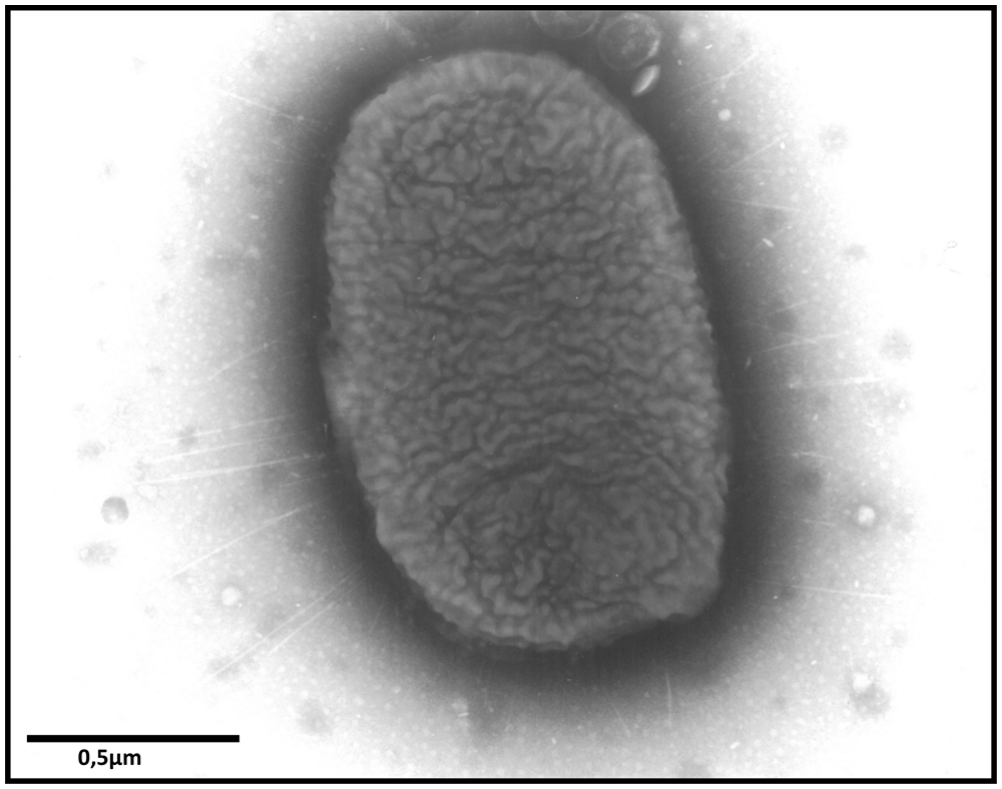
**Transmission electron microscopy image showing fimbriae of *S.* Enteritidis after culture on Sven Gard plates.** Bar represents 0.5 μm.

Fimbriae are proteinaceous surface appendages of 0.5–10 μm in length and 2–8 nm in width (**Figure 1**), which have, at their distal part, a protein which interacts with its host receptor thus mediating the adhesion of the bacteria to the host or inert surfaces. So far, more than 10 fimbrial operons have been identified in *Salmonella* genomes and the number and types of fimbrial operons depends on the serotype (van Asten and van Dijk, 2005). Horizontal gene transfer and deletion events have created unique combinations of fimbrial operons among *Salmonella* serotypes (Baumler et al., 1997; Townsend et al., 2001). The combination of adhesins used by each serotype affects its ability to adhere to different cell types and therefore contributes to the ability of this serotype to colonize different niches or hosts. Thirteen fimbrial operons have been identified in *S.* Typhimurium: *agf* (also called *csg*), *fim*, *pef*, *lpf*, *bcf*, *saf*, *stb*, *stc*, *std*, *stf*, *sth*, *sti*, and *stj*. Until now, studies of fimbriae have been slowed down by the fact that only one of them, the Type I fimbriae (also called Fim fimbriae or SEF21), is expressed in commonly used laboratory culture conditions. This can in part be related to a post-transcriptional control of other fimbrial gene expression via the 5′untranslated region of the *fimAICDH* transcript or to a negative control of their expression as observed for the *std* operon that is repressed by Dam, SeqA, HdfR, and RosE (Chessa et al., 2008a; Sterzenbach et al., 2013). There is, however, evidence that these adhesive structures can be expressed *in vivo*. Indeed, BcfA, FimA, LpfA, PefA, StbA, StcA, StdA, StfA, and StiA have been shown to be expressed after inoculation of bovine ileal loops with *S.* Typhimurium. Moreover, antibodies against the same fimbrial proteins and also against AgfA and SthA have been observed after inoculation of mice with *S.* Typhimurium (Humphries et al., 2003, 2005).

Due to the difficulties encountered to study fimbriae, their respective cell receptor and targeted cell types in their animal hosts are known for only a few of them. Type I fimbriae are characterized by hemagglutination, yeast agglutination, and binding to eukaryotic cells expressing the α-D-mannose receptor (Korhonen et al., 1980). Long polar fimbriae mediate the adhesion of *S.* Typhimurium to murine Peyer patches while Pef fimbriae, whose binding carbohydrate is the Lewis X blood group antigen, are involved in adhesion to murine villous small intestine (Baumler et al., 1996; Chessa et al., 2008b). Std fimbriae bind terminal Fucα1-2 moieties present in the mucus layer of the murine caecum mucosa or on the surface of cells such as Caco-2 cell line (Chessa et al., 2009) and thin aggregative fimbriae (also called Tafi or Curli), encoded by *agf* operon, interact with the extracellular matrix glycoproteins. While the interactions of fimbriae with animal cells are not well characterized, several studies have shown that fimbriae are involved in the colonization of different animals. Type I fimbriae contribute to mouse, pig, and chick intestinal colonization (Dibb-Fuller and Woodward, 2000; Althouse et al., 2003). *lpf*, *bcf*, *stb*, *stc*, *std*, and *sth* encoded fimbriae lead to the long-term persistence of *S.* Typhimurium in resistant mice (Nramp^+/+^) (Weening et al., 2005; Lawley et al., 2006). In chicks, *pef*, *std*, *sth*, *sef*, and *agf-*encoded fimbriae are also involved in spleen and intestinal colonization by *S.* Typhimurium and *S.* Gallinarum (Morgan et al., 2004; Shah et al., 2005). Usually, the absence of expression of only one fimbriae type does not greatly reduce *Salmonella* virulence. However, multiple mutations have a greater impact. For example, in *Salmonella* susceptible mice (Nramp^-/-^), a *S.* Typhimurium strain where the three *pef*, *lpf* and *agf* operons are deleted, has a 29-fold higher 50% lethal dose (LD_50_) and is less able to colonize the intestine than the wild-type strain or than strains with a single mutation after oral inoculation, thus highlighting the synergistic action of fimbriae to colonize the intestine (van der Velden et al., 1998).

Some fimbriae also contribute to biofilm formation in animals and plants, particularly curli fimbriae. These fimbriae are required for biofilm formation on epithelial cells and chicken intestinal surfaces and favor the attachment and the persistence of the biofilm-associated *Salmonella* on alfalfa sprouts, parsley, and tomato leaflets (Barak et al., 2005, 2007; Ledeboer et al., 2006; Jonas et al., 2007; Lapidot and Yaron, 2009; Cevallos-Cevallos et al., 2012). They also promote survival of *Salmonella* inside plants (Gu et al., 2011). A role of Pef and Lpf fimbriae has also been observed in biofilm formation on animal surfaces. As curli fimbriae, Pef fimbriae have been shown to be required for biofilm formation on inert, epithelial cells and chicken intestinal surfaces, while Lpf fimbriae appear to be more involved in biofilm formation on chicken intestinal tissue than on plastic or tissue culture cells (Ledeboer et al., 2006; Jonas et al., 2007). In addition to the curli fimbriae involved in plant colonization, fimbriae encoded by the *stf* operon have been shown to increase the persistence of *S.* Typhimurium on intact but not on damaged lettuce leaves after cold storage but this seems not to be related to an attachment defect on leaf tissue (Kroupitski et al., 2013).

#### Non-fimbrial adhesins

Two types of non-fimbrial adhesins have been described in *Salmonella* according to their secretion pathway: BapA and SiiE are each secreted by a Type-1 secretion system, while ShdA, MisL, and SadA are autotransporters also known as Type-V secretion systems.

BapA (386 kDa) and SiiE (595 kDa) are the largest proteins of *Salmonella* and share the characteristics of having numerous bacterial Immunoglobulin-like domains. The genes encoding these two proteins are highly conserved among *Salmonella* serotypes (Biswas et al., 2011; Suez et al., 2013). BapA has been shown to be involved in biofilm formation in *S.* Enteritidis. Its expression is co-regulated with the two other essential components of *Salmonella* biofilms, i.e., thin aggregative fimbriae and cellulose, by the central transcriptional regulator AgfD (Latasa et al., 2005). In mice, BapA is involved in the first steps of the infectious process as a *bapA S.* Enteritidis mutant was less able to colonize mice ileal loops than the wild-type strain and was shown to be less virulent for mice than its parent only when orally inoculated (Latasa et al., 2005). The role of BapA in *S.* Typhimurium is less clear (Latasa et al., 2005; Jonas et al., 2007). In plants, no studies have been performed, but the role of BapA in biofilm formation supports a possible role of this protein in *Salmonella/*plant interactions.

SiiE is an adhesin encoded by *Salmonella* Pathogenicity Island-4. The *siiABCDEF* operon encodes the adhesin and the proteins required for the biosynthesis of its Type-I secretion system. The SiiE protein mediates the initial adhesion of *Salmonella* to the apical side of polarized epithelial cells via multiple interactions with glycostructures with terminal *N*-acetyl-glucosamine and/or α 2,3-linked sialic acid. This SiiE-mediated adhesion is required for subsequent Type III-secretion-system-1 (T3SS-1) invasion of these cells (detailed in Section “T3SS-1 Dependent Mechanism”; Gerlach et al., 2008; Wagner et al., 2014). In line with the cooperation of SiiE and the T3SS-1, the *siiABCDEF* operon is co-regulated with the *Salmonella* Pathogenicity Island-1 (SPI-1) genes involved in T3SS-1 biosynthesis (Gerlach et al., 2007b; Main-Hester et al., 2008). Contrary to BapA, SiiE has not been shown to contribute to biofilm formation even if a role of SPI-4 in the virulence of *Salmonella* has been observed in animals (Latasa et al., 2005). Indeed, mutants in the *siiABCDEF* operon, including a *siiE* mutant, were attenuated for colonization of mice after oral but not after intraperitoneal infection compared to their wild-type parents (Morgan et al., 2004; Kiss et al., 2007). However, no attenuation was observed by Gerlach et al. (2007a) in a similar model. The role of this giant adhesin in plant colonization remains to be determined.

The ShdA adhesin is a monomeric fibronectin and collagen-I binding protein that is encoded by *shdA* carried on the CS54 island (Kingsley et al., 2004). This gene is present in *Salmonella* serotypes isolated from human and warm-blooded animals but not from cold-blooded animals (Kingsley et al., 2000). ShdA was shown to mediate adhesion to the epithelium of the murine caecum (Kingsley et al., 2002) and to contribute to the colonization of this organ and of the Peyer’s patches of the terminal ileum of mice. A *shdA* mutant also had a reduced persistence in the cecum and a fecal shedding defect in this animal model but not in a pig model (Kingsley and Baumler, 2000; Kingsley et al., 2003; Boyen et al., 2006). No data on the role of ShdA in plant colonization is available.

The MisL adhesin, encoded by a gene within SPI-3, shares several characteristics with ShdA. MisL is a monomeric adhesin that is not expressed under standard *in vitro* cultures but its expression can be induced by the transcriptional regulator MarT encoded on SPI-3 (Blanc-Potard et al., 1999; Tükel et al., 2007). In addition, as ShdA, MisL binds fibronectin and is involved in the colonization of the cecum and in the persistence of *S.* Typhimurium in mice after oral inoculation (Dorsey et al., 2005). A *misL* mutant has also been shown to be altered in the intestinal colonization of chicks and in the attachment to lettuce leaves (Morgan et al., 2004; Kroupitski et al., 2013). The latter phenotype could be related to a reduced ability of the mutant to form biofilms on inert surfaces (Kroupitski et al., 2013).

Little is known about the trimeric SadA adhesin. Contrary to ShdA and MisL, this protein is expressed under *in vitro* standard growth cultures and is surface-exposed on *S.* Typhimurium. Its expression on bacterial cells deprived of *O*-antigen mediates autoaggregation and biofilm formation on inert surfaces. Moreover, SadA has been shown to increase the adherence and invasion of an *Escherichia coli* strain lacking smooth lipopolysaccharide (LPS) into human intestinal Caco-2 cells. However, no binding with extracellular matrix molecules collagen I, collagen III, collagen IV, elastin, fibronectin, and laminin has been observed and no role of SadA in the virulence of *S.* Typhimurium in mice and in *C. elegans* models has been demonstrated (Raghunathan et al., 2011).

#### Other structures

Flagella and LPS are bacterial factors whose main function is not to mediate adhesion. Flagella confer motility and chemotaxis and stimulate the host innate immune response (Vijay-Kumar and Gewirtz, 2009). LPS is a major component of the outer membrane of most Gram-negative bacteria, and protects them from toxic compounds, such as antibiotics or bile salts. LPS is composed of three parts: the lipid A, which is embedded in the bacterial membrane, the core oligosaccharide, and the most external moiety, the *O*-antigen. It is also an endotoxin responsible for septic shock in animal hosts and, as flagella it stimulates the innate immune response (Tan and Kagan, 2014). However, several papers describe a role of these structures in the adhesion of *Salmonella* to animal or plant tissues. For flagella, the reduced adhesion of *Salmonella* described in some papers in animal models is related to a defect in the motility function conferred by flagella (Jones et al., 1981; Khoramian-Falsafi et al., 1990). This could be explained by the fact that a strain with reduced motility is less likely to enter in contact with its target host cells/tissues and consequently has a reduced attachment/entry rate into cells. However, in other papers, flagella, *per se*, were shown to be involved in adhesion, as mutants in flagellar structure proteins were shown to be impaired in adhesion to chick gut explants and in biofilm formation on cholesterol-coated surfaces, unlike paralyzed mutants (Allen-Vercoe and Woodward, 1999; Crawford et al., 2010). In plants, a role of flagella in the adhesion to basil and lettuce leaves has also been reported (Berger et al., 2009; Kroupitski et al., 2009). In addition, it is important to note that two open reading frames involved in swarming motility are also involved in plant colonization (Barak et al., 2009).

A few papers describe a role of the LPS in the adhesion of some *Salmonella* serotypes. Indeed, *S.* Choleraesuis and *S.* Typhi, rough mutants, i.e., with an *O-*antigen defect, were altered in the attachment and invasion of polarized epithelial monolayers of Madin Darby canine kidney (MDCK) cells and HeLa cell monolayers, respectively (Finlay et al., 1988; Mroczenski-Wildey et al., 1989). However, the absence of *O*-antigen expression was shown to have the opposite effect in *S.* Typhimurium and *S.* Enteritidis serotypes (Kihlstrom and Edebo, 1976; Baloda et al., 1988). In the latter case, the strongest ability of rough mutants to adhere to eukaryotic cells was suggested to be related to the highest hydrophobicity properties of these mutants compared to their wild-type parents, thus allowing hydrophobic interactions between the bacterial and the host cell membranes. In plants, the *O*-antigen capsule was shown to be involved in the colonization of alfalfa sprouts, while colonic acid, another extracellular polysaccharide, was not. Indeed, a mutant defective in the assembly and translocation of the *O*-antigen capsule had a reduced ability to adhere to alfalfa sprouts (Barak et al., 2007). However, *O*-antigen capsule production did not confer a selective advantage to *S.* Typhimurium for red ripe tomatoe colonization (Noel et al., 2010).

As mentioned above, biofilm formation is an important property for *Salmonella* adhesion to plants. In line with this, cellulose, which is the main exopolysaccharide of the biofilm matrix, is involved in the adhesion and colonization to/of lettuce and parsley leaves and alfalfa sprouts (Barak et al., 2007; Lapidot and Yaron, 2009; Kroupitski et al., 2013).

Most *Salmonella* adhesive structures are expressed only *in vivo* thus rendering difficult their study. Even if the constitutive expression of these surface components and the study of the regulation of their expression have promoted *in vitro* studies in the last few years, much work is still required to understand the role of each of them and their potential cooperation and/or redundancy in mediating *Salmonella* interaction with their hosts.

### INVASION

In animals, *Salmonella* has developed different mechanisms to induce its own internalization in different cell types in order to survive, multiply, and spread through the host (Rosselin et al., 2012). Until recently, it was assumed that *Salmonella* could enter cells using its T3SS-1(Ibarra and Steele-Mortimer, 2009). However, recent research has shown that *Salmonella* infection may occur independently of the T3SS-1 (Rosselin et al., 2011). While the internalization of *Salmonella* is demonstrated in animal cells, the presence of *Salmonella* inside plant cells remains controversial.

*Salmonella* have been found inside different plant tissues and even in the seeds inside fruits (Klerks et al., 2007a; Schikora et al., 2008; Gu et al., 2011). In addition, it has been shown that *Salmonella* is able to move within plants (Gu et al., 2011, 2013a,b). Several leaf structures have been postulated as the possible entry sites of *S.* Typhimurium (Kroupitski et al., 2009; Barak et al., 2011; Golberg et al., 2011). One report suggests that the trichomes are preferential colonization sites (Iniguez et al., 2005). However, Kroupitski et al. (2009) have shown that the preferential sites for *Salmonella* entry are the stomata, a natural opening on the leaf surface. Moreover, it has been postulated that this process depends on flagella. In addition, light seems to be required for *Salmonella* to move toward stomatal openings, because an artificial opening of the stomata in the dark had no effect on *Salmonella* internalization. Whether *Salmonella* is able to enter plant cells is still controversial. However, two laboratories observed intracellular localization of *Salmonella*: *S*. Typhimurium bacteria were observed inside rhizodermal cells of *Arabidopsis thaliana* and were shown to enter protoplasts of *Nicotiana tabacum* cells *in vitro*, although at a relatively low level (Samelis et al., 2003; Schikora et al., 2008). In addition, *S*. Typhimurium has been recovered from both lettuce leaves and surface-sterilized parsley leaves, supporting the hypothesis that *Salmonella* is able to invade the inner layers of leaf tissue (Franz et al., 2007; Kisluk and Yaron, 2012). However, in the latter case, the bacterial localization in plant cells was not demonstrated and requires more study.

#### T3SS-1 dependent mechanism

**FIGURE 2:**
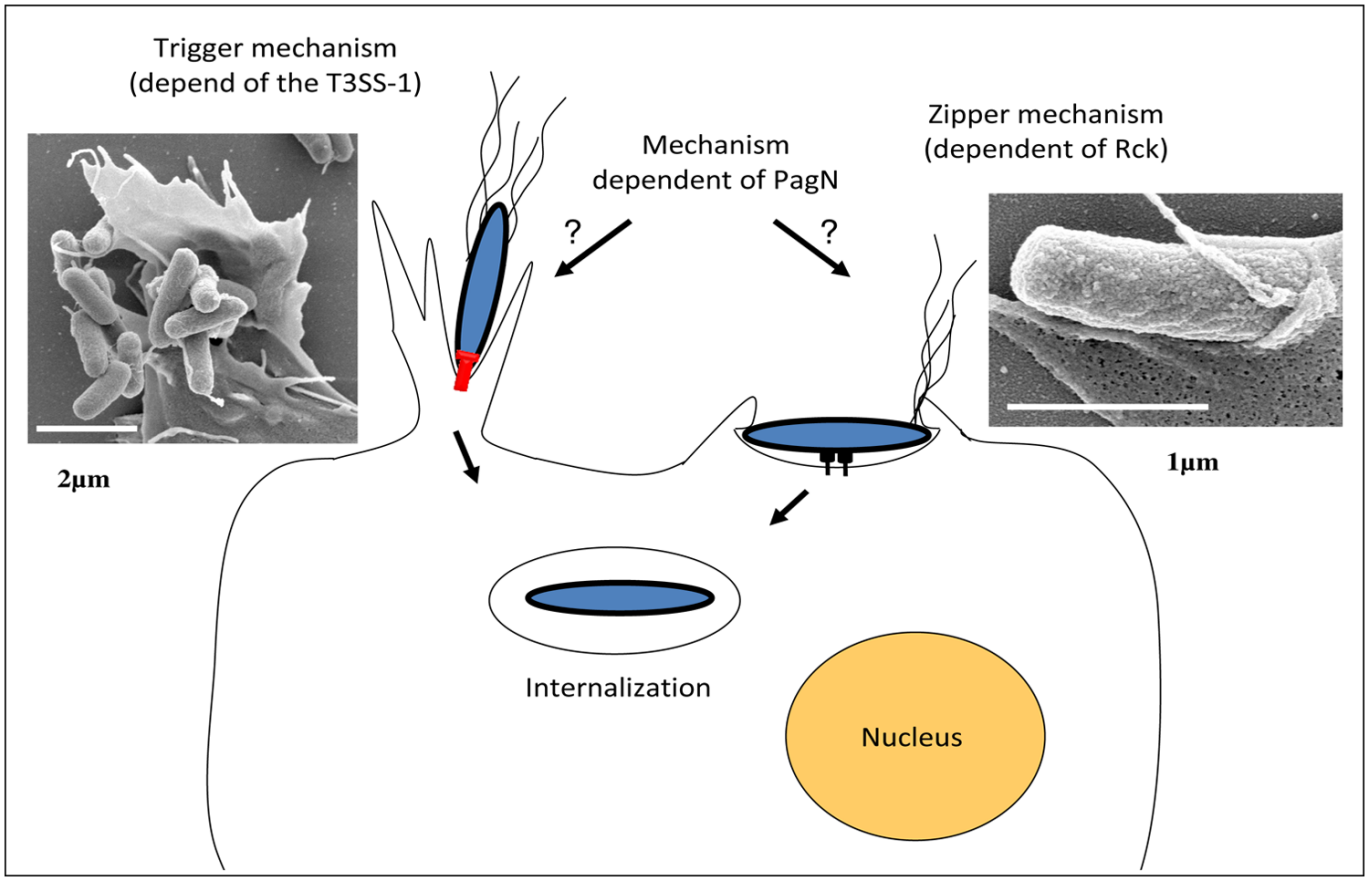
**Models of *Salmonella* invasion mechanisms.** *Salmonella* uses T3SS-1 to translocate effector proteins directly into host cells (left side; Bar of the transmission electron microscopy image represents 2 μm). Several of these effector proteins modulate host cell actin cytoskeleton, leading to an intense membrane ruﬄing and internalization of the bacteria into a modified phagosome or *Salmonella*-containing-vacuole (SCV). *Salmonella* can also invade cells via a T3SS-1-independent mechanism, which is induced by the *Salmonella* Rck membrane protein interacting with its receptor on the host cell plasma membrane and characterized by the induction of thin membrane extensions (right side; Bar of the transmission electron microscopy picture represents 1 μm). The membrane rearrangements induced by the *Salmonella* invasin PagN have not been studied yet.

The SPI-1 island encodes structural components of the secretory machinery, chaperones, regulators, and some effectors involved during mammalian host invasion. When *Salmonella* reaches the intestinal environment, the SPI-1 genes are expressed, allowing assembly of the T3SS-1 at the bacterial surface (Kubori et al., 1998). After an interaction between the host cell and the bacteria the T3SS-1 translocates into host cells at least 15 proteins encoded within the SPI-1, SPI-5 pathogenicity islands, and pro-phages (Garner et al., 2002; Hayward et al., 2005; McGhie et al., 2009). Among these effectors, SopE, SopE2, SopB, SipA, SipC, and SptP have been shown to be required for cell invasion by *Salmonella*. The synergistic activity of SopE, SopE2, SopB, SipA, and SipC induces actin recruitment and polymerization at the entry site, which results in the formation of “ruﬄes” at the membrane surface (**Figure 2**; McGhie et al., 2009). These ruﬄes extend from the cell surface and internalize the bacteria in the host cell in a vacuole. After ruﬄe formation, the endocytic vacuole closes and the cellular cytoskeleton of the host cell returns to its initial state, allowing the cell to return to its original morphology (Fu and Galán, 1999). The effector SptP allows this restoration by reorganizing the actin cytoskeleton (Fu and Galán, 1998, 1999). This T3SS-1 invasion process is referred to as a “Trigger mechanism” and has only been studied in mammalian cells (Velge et al., 2012).

Nevertheless, T3SS-1 contribution to *Salmonella* pathogenesis depends on the model used. In bovine, rabbit, and murine models, the T3SS-1 of *S.* Dublin and *S.* Typhimurium serotypes is essential for intestinal colonization (Wallis and Galyov, 2000). However, some *Salmonella* lacking the T3SS-1 remain pathogenic in different *in vivo* infection models such as a SPI-1 mutant of *S.* Gallinarum in adult chicken (Jones et al., 2001) or *S.* Typhimurium and *S.* Enteritidis mutants in one week-old chicks or Balb/C mice (Coombes et al., 2005; Jones et al., 2007; Karasova et al., 2010). Furthermore, *S.* Seftenberg strains lacking SPI-1 have been isolated from human clinical cases, suggesting that for this serotype, the T3SS-1 is not required to establish infection in humans (Hu et al., 2008).

Interestingly, reducing the virulence of *Salmonella* by removing T3SS-1 increased colonization of alfalfa roots and wheat seedlings (Iniguez et al., 2005). However, these results contrast with the reduced proliferation observed for *prgH* mutants, lacking a functional T3SS-1, in *A. thaliana* (Schikora et al., 2011). In addition, more apparent symptoms in *Arabidopsis* plants are observed with these mutants, suggesting that, in this case, the hypersensitive response (HR) seems to be prevented by the effectors secreted by the T3SS-1 (detailed in Section “Host Defenses”). Overall, this suggests that the T3SS-1-dependent successful colonization seems to be plant-species-specific and that *Salmonella* strains may have different pathogenicity toward plants.

The role of the T3SS-1 in *Salmonella*–plant interactions raises many questions concerning the signals which induce expression of the T3SS-1 in plants and the mechanisms set up by *Salmonella* to deliver effectors.

#### T3SS-1 independent mechanisms

To date, two *Salmonella* invasins called Rck and PagN have been identified. Moreover, studies have revealed that invasion systems of *Salmonella* are not restricted to the PagN, Rck, and T3SS-1. A *Salmonella* mutant unable to express the T3SS-1, Rck, or PagN was indeed still able to enter different animal cell lines (Rosselin et al., 2012).

***Rck invasin.*** Rck invasin is encoded by the *rck* gene located on the *S.* Enteritidis and *S.* Typhimurium large virulence plasmid (Rotger and Casadesús, 1999). Rck belongs to a family of outer membrane proteins (OMP) associated with virulence functions, including PagC which is involved in *Salmonella* intracellular survival and Ail, a *Yersinia* invasin (Heffernan et al., 1992). The role of Rck in the invasion of *Salmonella* in animal cells has been well described *in vitro* and demonstrated through different methods. Rosselin et al. (2010) have shown that *rck* deletion in *S.* Enteritidis leads to more than a twofold decrease in animal epithelial cell invasion without altering the bacteria attachment to the cells. In addition, it has been shown that Rck alone is able to trigger cell invasion in a receptor-dependent manner by using Rck-coated latex beads and initially non-invasive *E. coli* strain overexpressing Rck. The minimal region of Rck required to induce invasion corresponds to the G113-V159 peptide. At the cellular level, the interaction of Rck with its receptor expressed on an animal cell membrane leads to a signaling cascade, involving cellular proteins which promote local accumulation of actin and weak and closely adherent membrane extensions. This process is referred to as a “Zipper” mechanism and has only been studied in animal cells (**Figure 2**; Rosselin et al., 2010).

However, in animal *Salmonella* pathogenesis, the role of Rck is still poorly understood. The regulation of Rck regulated by quorum sensing via SdiA, suggests that Rck may play an intestinal role (Ahmer et al., 1998). Dyszel et al. (2010) have reported that Rck confers a selective advantage for intestinal colonization in mice when it is expressed. Moreover, as *rck* is regulated by an unidentified system, which is independent of SdiA at 37 and 42°C (Smith et al., 2008), it is conceivable that Rck has a role which is not only restricted to the gastrointestinal tract and which could be induced in only some animal species.

The role of quorum sensing in *Salmonella* pathogenesis in animals and its impact on Rck expression is still poorly characterized. However, in plants, the quorum sensing which allows plant pathogen colonization of rhizosphere and phyllosphere has been well documented (Daniels et al., 2004; Dulla and Lindow, 2009). A study of the possible role of Rck in plant colonization is ongoing.

***PagN invasin.*** In addition to Rck and the T3SS-1, an OMP called PagN is involved in *Salmonella* animal host invasion (Lambert and Smith, 2008). *PagN* is similar to both the Hek and Tia invasion proteins of *E. coli*. This OMP is encoded by the *pagN* gene, which is located on the centisome 7 genomic island. PagN protein is widely expressed among the different *Salmonella enterica* serotypes (Folkesson et al., 1999). Lambert and Smith (2008) have shown that the deletion of *pagN* in *S.* Typhimurium leads to a significant decrease in animal cell line invasion without altering the bacteria-cell adhesion. In addition, expression of PagN in a non-invasive *E. coli* strain resulted in adhesion to and invasion of animal cell lines. At the cellular level, it was shown that PagN-dependent invasion requires an interaction of PagN with the cell surface heparin sulfate proteoglycans, which could lead to actin polymerization at the entry site (Lambert and Smith, 2008, 2009). However, the membrane proteoglycans are diverse and only a few membrane proteoglycans can transduce a signaling cascade. Another hypothesis is that they could play a role as a co-receptor for invasion and not as a receptor itself.

In a mouse model, it has been shown that PagN is required for *Salmonella* survival (Heithoff et al., 1999) and that spleen colonization of a *pagN* mutant is lower than that of its parental strain (Conner et al., 1998). However, the precise role of PagN in *Salmonella* animal and plant pathogenesis remains unknown. The PhoP/PhoQ two-component regulatory system activates *pagN*, leading to a maximal expression under the conditions found in the intracellular *Salmonella*-containing vacuole (SCV), which is known to downregulate T3SS-1 expression (Conner et al., 1998; Heithoff et al., 1999; Eriksson et al., 2003). Thus, *Salmonella* could express a high level of PagN when the bacteria exit the SCV and the cell, which may facilitate interactions with other cells that the pathogen encounters (Lambert and Smith, 2008). However, the role of PagN in plants remains to be studied.

***Non-identified invasion factors.*** In animals, recent research has shown that invasion factors in *S.* Enteritidis and *S.* Typhimurium are not limited to PagN, Rck, and the T3SS-1. Rosselin et al. (2011) have demonstrated that a strain which does not express the T3SS-1, PagN, or Rck, is still able to significantly invade some animal cells. This idea is reinforced by the study performed by Aiastui et al. (2010) and van Sorge et al. (2011) who showed that a *Salmonella* strain lacking the T3SS-1 which does not express PagN and Rck, was still able to enter different cell types (epithelial, endothelial, and fibroblasts cells). In addition, *S.* Typhimurium invasion studies of a 3-D intestinal epithelium have also supported the idea that *Salmonella* expresses invasion factors, which have not yet been characterized (Radtke et al., 2011).

### MULTIPLICATION

Once internalized into the tissue, *S.* Typhimurium is able to multiply. The ability to colonize plants may be an effective survival and multiplication strategy for *Salmonella* as it provides a link between its excretion in the environment via animal feces and the recontamination of herbivorous and omnivorous hosts. Many studies have been conducted on the behavior and multiplication of *Salmonella* in animal hosts, some on plants, especially on the foliage of plants, but very few have been conducted within plant cells (Barak and Liang, 2008). *Salmonella* can also multiply in the rhizosphere (Semenov et al., 2009).

#### The different multiplication areas of Salmonella

In order to effectively colonize plants, bacteria need to grow and spread. Growth requires bacteria to either synthesize indispensable metabolites or acquire essential nutrients from their environment. *Salmonella* is unable to liberate nutrients from plant cells as plant pathogens do because they lack enzymes to degrade plant cell walls (Teplitski et al., 2009). However, they often grow using nutrients liberated by plant cell lysates and root exudates after action of plant pathogens (Barak and Schroeder, 2012). In this context, *Salmonella* has to adapt to both the plant phyllosphere and rhizosphere, which are heterogeneous environments varying in physical conditions and nutrient availability (Barak and Schroeder, 2012). The leaf surface is, for example, a harsh environment for bacteria due to UV radiation, the heterogeneity of nutrient availability and rapid fluctuations in temperature, and free water availability. However, plant surfaces are not homogenous and contain various microsites that represent oases of available nutrients and which may support multiplication of human pathogens after contamination events (Brandl et al., 2013). Indeed, *Salmonella* has been shown to preferentially move on leaves toward open stomata and colonize the vein areas, the bases of trichomes and damaged leaf areas, which may provide shelter and increase nutrient and water availability (Monier and Lindow, 2005). In addition, inoculation of leaves with *S.* Typhimurium can result in contamination of tomato fruit through internal movement of the bacteria from leaves into the fruit (Gu et al., 2011).

*Salmonella* appear to be successful secondary colonists, benefiting from the action of phytopathogens, e.g., suppression of plant defenses and plant tissue damage (lesions, water soaking, and soft rots). Numerous studies have shown that soft-rot bacteria promote proliferation of *Salmonella* in plants. Biotrophic plant pathogens, like *P. syringae* and *Xanthomonas campestris*, can promote growth or survival of *Salmonella* and enterohaemorrhagic *E. coli* on plants (Barak and Liang, 2008; Aruscavage et al., 2010; Potnis et al., 2014). Formation of lesions on leaves by both these phytopathogens has been associated with an increase availability of total sugars, specifically, innositol and sucrose (Aruscavage et al., 2010). Moreover, *Salmonella* can benefit from the immune-suppressing action of plant pathogenic bacteria like *Pseudomonas syringae* pv. tomato (Meng et al., 2013) and *Xanthomonas perforans*, which suppress the pathogen-associated molecular pattern (PAMP)-triggered immunity (Potnis et al., 2014). *Salmonella* found in preexisting plant bacteria biofilms was more likely to survive dry conditions on lettuce and cilantro leaves than solitary bacteria (Rastogi et al., 2012). These observations suggest that *Salmonella* may find refuge not only in particular physical microsites on plants but also in microbial conglomerates where protection from adverse conditions outweighs potential competition and antibiosis from other plant colonists. For example, Goudeau et al. (2013) observed that population sizes of *S.* Typhimurium increased 56-fold when inoculated alone onto cilantro leaves, compared to more than 2,800-fold when co-inoculated with *Dickeya dadantii*, a prevalent pathogen that macerates plant tissue. The global gene expression profile of *Salmonella* in soft-rotted tissue showed that there was a lack of competition for nutrients between these two bacterial species due to resource partitioning. Moreover, 29% of the genes that were upregulated in cilantro macerates had also previously been observed to have increased expression levels in the chicken intestine (Goudeau et al., 2013). Commonalities between soft rot lesions and the intestine such as anaerobic conditions and nutritional resources indicate an important overlap in the ecological niche and may explain the adaptation of *Salmonella* to both kingdoms (Goudeau et al., 2013).

The gastrointestinal tract represents a vast mucosal surface vulnerable to attack by enteropathogens. It is fortified with a variety of physical and immunological defense barriers. The colonizing microbiota represents a major protective shield. This dense population is thought to provide both a physical barrier for the attachment of bacterial pathogens to surfaces, and to compete for essential nutrients (Caricilli et al., 2014). The microbiota is also able to produce a nutritional environment unfavorable to growth of bacterial pathogens. This protective mechanism has been termed “colonization resistance” and helps to prevent infection (Van Immerseel et al., 2005). In addition to colonization resistance, the microbiota mediates *S*. Typhimurium clearance from the gut lumen (Endt et al., 2010). However, other reports have shown that *Salmonella* uses ingenious mechanisms to hijack the mucosal inflammation for its own benefit, with detrimental effects for the host and the microbiota (Fabrega and Vila, 2013). For example, using the T3SS virulence factors, *S*. Typhimurium is able to elicit a host inflammatory response, which ultimately helps the pathogen. The intestinal microbiota produces hydrogen sulfide, which normally becomes detoxified to thiosulphate by host cells. The inflammatory response, induced by *Salmonella*, leads to the migration of neutrophils into the intestinal lumen and the subsequent release of reactive oxygen species (ROS). When thiosulphate is exposed to ROS it is oxidized to tetrathionate, which can be used by *S*. Typhimurium as an alternative electron acceptor. Thus the utilization of tetrathionate as a terminal electron acceptor in respiration is a far more efficient process for energy generation than fermentation used by anaerobic microbiota (Winter et al., 2010). This respiratory pathway allows *S.* Typhimurium to use ethanolamine, which does not support growth of intestinal microbiota (Thiennimitr et al., 2011). Thus inflammation leads to a marked boost in *S*. Typhimurium growth.

Similar to the protective role of microbiota in intestinal tract, plants have protective microbial communities. In the rhizosphere, plant growth-promoting bacteria fend off invaders by activating the induced systemic resistance (ISR) response in plants, through the production of antibiotics and competition for nutrients and iron (Pieterse et al., 2014; Sang et al., 2014). Within plants, endophytic bacteria also defend the plant against pathogens. Gu et al. (2013a) have suggested that invasion of tomato plants by *S.* Typhimurium is inversely correlated to the diversity of endophytic bacteria.

Besides the mechanisms of metabolic cooperation or competition between plant or intestine microbiota and *Salmonella*, cell-to-cell signaling in multispecies microbial communities plays an important role in both plants and gut habitats. The contribution of signaling via quorum sensing circuits mediated by either *N*-acyl homoserine lactones (AHL) or the autoinducer-2 (AI-2) to the behavior of *Salmonella* in plant-associated bacterial communities and in animal intestines has already been demonstrated (Ahmer and Gunn, 2011; Brandl et al., 2013). However, the importance of AHL and AI-2-based signaling in *Salmonella* during the interactions of *Salmonella* both with plant and animal bacteria requires further investigation (Thomanek et al., 2013).

Proliferation of *Salmonella* in some plant tissues has been reported to cause disease-like symptoms. In *Arabidopsis*, immersion of seedlings in a dense suspension of *Salmonella* or infiltration of leaves with the pathogen can elicit chlorosis, wilting, or tissue necrosis (Schikora et al., 2008; Berger et al., 2011). The symptoms elicited by *Salmonella* were related to the presence of SPI-1 and SPI-2, which also play a key role in host animal infection (Schikora et al., 2011). Generally, it was believed that *Salmonella* survived on plant tissues after contact with contaminated water or animal manures. However, endophytically present *Salmonella* was observed in the vascular system of *S.* Typhimurium-inoculated tomato leaves (Gu et al., 2011). Moreover, *Salmonella* was observed intracellularly in *A. thaliana* protoplasts and in cultured tobacco cells (Schikora et al., 2008; Shirron and Yaron, 2011). However, very little is known on the intracellular multiplication mechanisms in plant cells. It has been shown that several T3SS *Salmonella* mutants have reduced proliferation in plants, compared to the wild-type strain (Schikora et al., 2011). The same study demonstrated that symptoms caused by the T3SS mutants in *Arabidopsis* plants were more pronounced, suggesting that plants can react to *Salmonella* infection with a HR and that T3SS mutants were unable to hamper the induced HR (Schikora et al., 2011). In animals, numerous studies have analyzed the multiplication mechanisms at the cell level and especially the role of the T3SS-2 in intracellular multiplication of *Salmonella* Typhimurium.

#### Intracellular multiplication within animal cells

**FIGURE 3:**
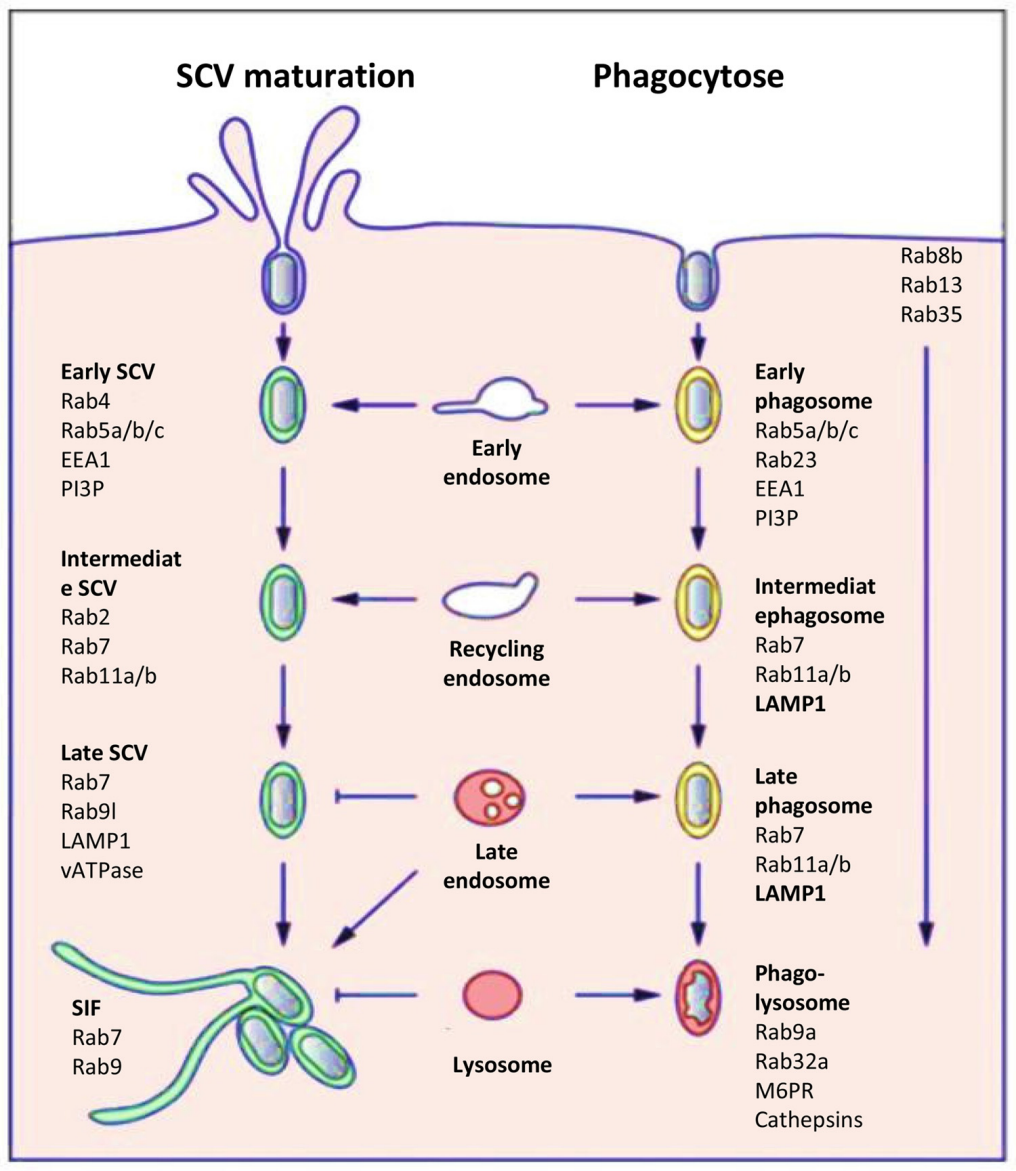
**Host cell markers present on the SCV (**left**) or on a phagosome (**right**).** Comparison of the host cell markers, which characterize the classic endosome process and the biogenesis and maturation of the SCV (Figure modified from Bakowski et al., 2008).

*Salmonella* can enter host cells through its T3SS-1 or to its Rck and PagN invasins (detailed in Section “T3SS-1 Independent Mechanisms”). However, unlike for the T3SS-mediated entry process, no studies have examined the intracellular behavior of *Salmonella* internalized in animal or plant cells via the invasin-mediated processes. Following entry in host cells, thanks to the T3SS-1, the majority of *Salmonella* resides in a membrane-bound compartment known as the SCV. Biogenesis and maturation of the SCV has been extensively studied in many cell types and mainly for *S.* Typhimurium (Bakowski et al., 2008; Figueira and Holden, 2012; Fabrega and Vila, 2013). The SCV, which allows bacterial growth, is distinct from a classical phagosome (**Figure 3**). *S.* Typhimurium in the SCV delivers into the host cell cytosol more than 30 effectors encoded by different *Salmonella* pathogenicity islands or the large virulence plasmid using a second type three secretion system called T3SS-2 (Fabrega and Vila, 2013). The T3SS-1, with its associated effectors, is expressed early, and is critical for cell invasion, early SCV biogenesis and the intestinal phase of infection and in particular induction of inflammation (Lostroh and Lee, 2001). The T3SS-2 is expressed a few hours following entry into cells and is responsible through effectors for SCV maturation, intracellular bacterial survival and the systemic phase of infection (Hensel, 2000). Improved understanding of these two secretion systems and of the interplay between effectors translocated by each T3SS has shown that their roles are not so clearly separated. For *S.* Typhimurium, it has recently been shown that the T3SS-2 play a role during the intestinal phase of infection, while the T3SS-1 translocated effectors also act in late stages of intracellular multiplication and SCV maturation (Bakowski et al., 2008). The full repertoire of T3SS-2 effectors is not present in all *Salmonella enterica* serotypes. However, loss of function of the T3SS-2 in different serotypes induces a strong virulence defect characterized by an intracellular growth defect or a loss of systemic infection ability.

Once internalized, the next event triggered by *Salmonella* is the maintenance of the SCV by preventing delivery of antimicrobial host factors such as proteases and free-radical-generating complexes and by remodeling the organization of the host cell cytoskeleton to impair vesicular transport (Rajashekar and Hensel, 2011). The SCV is considered as a unique organelle that diverts from the normal endocytic pathway and allows *Salmonella* to survival and replication intracellularly. Numerous host cell markers associated with this endocytic pathway have been identified and the pattern of recruitment/retention of individual cell markers on the SCV induced by a virulent *Salmonella* strain is in part distinct from that of the classic model of phagosome, which for example, contains a non-virulent bacteria which is degraded in the phagolysosomes (**Figure 3**; Bakowski et al., 2008).

Compartments containing phagocytized material initially appear as early endosomes with markers such as early embryonic antigen 1 (EEA1), ARF6, Rab4, the transferrin receptor, and Rab5a and Rab5b GTPases (Smith et al., 2005). The maturation continues with the loss of early endosome markers and acquisition of late endosome markers like lysosomal glycoproteins (lpgs) such as LAMP1 and Rab7, Rab11a GTPases. The default maturation of phagosomes progresses toward the phago-lysosomes with the presence of lpgs, the mannose-6-phosphate receptor (M6PR), Rab9a, and Rab32a. Acquisition of vATPase on phago-lysosomes results in continuous acidification of the phagosomal vacuole. Through interaction with lysosomes, hydrolytic enzymes, in particular cathepsins are delivered into the vacuole and enzymatic activity results in the killing and degradation of internalized non-pathogenic bacteria. This maturation is usually completed within a time frame of two to three hours. To a certain extent, the SCVs show similar maturation and are initially integrated within the early endocytic pathway (Drecktrah et al., 2007). However, the compartments appear arrested in the late endosomal state with some features of late endosomes. They have an acidified lumen and express lysosomal membrane glycoproteins such as LAMP1, but the SCVs are not enriched in lysosomal hydrolases and thus do not express the M6PR, which delivers lysosomal hydrolases to the endosomal system (Steele-Mortimer et al., 1999). *Salmonella* T3SS-2 effectors trigger these modifications in host endocytic trafficking and functions in order to avoid complete fusion with secondary lysosomes. Here, the delivery of T3SS-2 effectors to the host cell cytosol is a precisely controlled process (Figueira and Holden, 2012). Two T3SS-2-related effectors, SigD and SpiC, have been reported to interact with this cell endocytic trafficking to escape from the classic degradation pathway (Uchiya et al., 1999). Moreover, by interacting with host cell proteins, SifA has been reported to compete in binding with Rab9, a small GTPase involved in modulation of cell endocytic trafficking (Jackson et al., 2008). Several hours after bacterial uptake, *Salmonella* induces *de novo* formation of an *F*-actin meshwork around bacterial vacuoles. This process is termed vacuole-associated actin polymerization (VAP) and is important to maintain the integrity of the SCV membrane (Meresse et al., 2001). Different experiments have revealed that not only the T3SS-2-dependent effectors SspH2, SseI, and SpvB but also the T3SS-1 effector SipA are involved in this process (Brawn et al., 2007). As the SCV matures and is surrounded by actin, it migrates toward a perinuclear position, which depends on the balanced activity of two microtubule proteins controlling microtubule formation: kinesin and dynein. This movement occurs, indeed, along microtubules in the direction of the microtubule-organizing centre (MTOC), where Golgi stacks accumulate (Ramsden et al., 2007). This position could allow acquisition of nutrients and membranes. Once SCV is correctly positioned, bacteria start replicating and initiate formation of *Salmonella* induced filaments (SIF) which are driven by the T3SS-2 effectors SifA, SipA, SseF, SseG and SseJ, in balance with the action of other effectors like PipB2 and SpvB (Fabrega and Vila, 2013). This process could control the integrity of the SCV membrane and its expansion, which is necessary for bacterial cell division. It is also possible that by controlling vesicular fusion on the SCV, these bacterial proteins ensure delivery of nutrients to the SCV, thereby facilitating bacterial replication.

The phenotypes and biochemical activity of several effectors reveal that their apparently opposing activities actually work together to control SCV membrane dynamics. It is thus remarkable that selective pressure and convergent evolution have triggered T3SS effectors to interfere both positively and negatively with the two major forms of post-translational modifications within eukaryotic cells: ubiquitination (SspH1, SspH2, SlrP)/deubiquitination (SseL), and phosphorylation (SteC)/dephosphorylation (SpvC) (Figueira and Holden, 2012).

#### Heterogeneity of Salmonella behavior within animal cells

**FIGURE 4:**
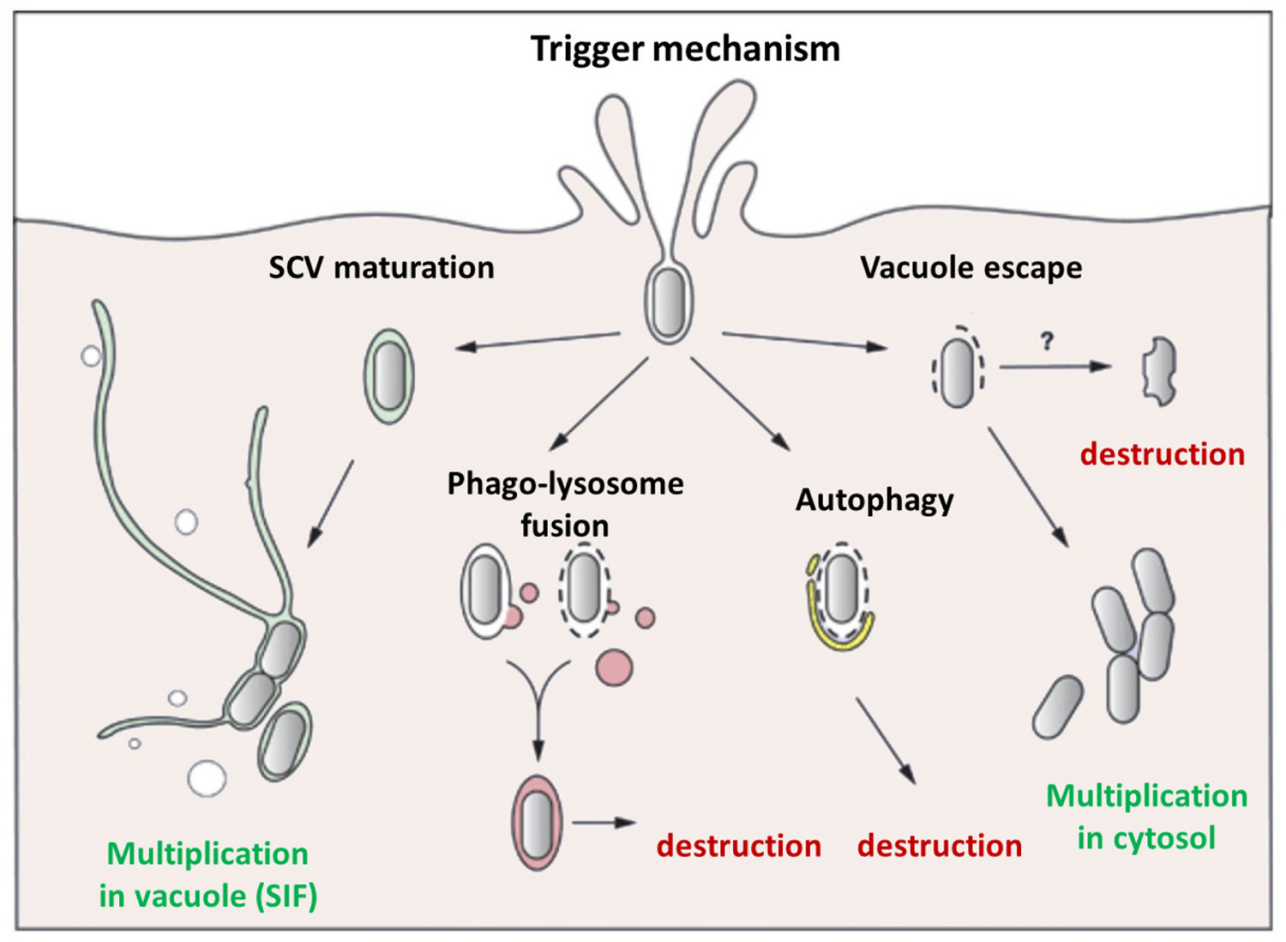
**Different behaviors of internalized *Salmonella*.** The majority of *Salmonella* strains, internalized within an animal cell by the Trigger mechanism mediated by the T3SS-1, are enclosed in a canonical SCV where they can multiply, and form SIF, which allow delivery of nutrients. However, in some cases the bacteria do not have the time (or the capability) to modify the vacuole leading to the fusion of the SCV with phago-lysosome triggering intra-vacuole destruction or autophagy. In other cases, *Salmonella* damages the SCV membrane triggering vacuole destruction, allowing bacteria to escape into the cytosol, where they can be destroyed, particularly in activated macrophages, or multiply extensively especially in epithelial cells. No data have been obtained for the Zipper mechanism induced by Rck.

The analysis of bacterial invasion process in animal host cells has revealed that intracellular *S.* Typhimurium populations are heterogeneous. The majority of bacteria reside in SCV which mature into replicative compartments. However, a fraction of the intracellular *Salmonella* encounters different fates, which seem to be controlled by different SCV maturations (**Figure 4**). Although *S.* Typhimurium generally excludes markers of mature lysosomes from the SCV, a few SCVs do acquire them. Indeed the protein hydrolase cathepsin D, and the fluid-phase marker-labeled lysosomes have been found to associate with a small fraction of intracellular bacteria (Garvis et al., 2001). *S.* Typhimurium in lysosome marker positive SCV seems to fail to overcome host cell defenses, leading to SCV–lysosome fusion and bacterial killing (Bakowski et al., 2008). However, data acquired by live-cell imaging in HeLa cells and using dextran as a general marker of the lysosomal compartment, showed that the classic SCV interacts with the endosomal system and associates with lysosomes without inducing death of bacteria (Drecktrah et al., 2007). These differences could also be related to SCV membrane damage, which could induce: (i) SCV–lysosome fusion (Viboud and Bliska, 2001), (ii) autophagy, a mechanism of capture of either cytosol-adapted or vacuolar bacteria which redirect them to the lysosomal compartment for killing (Knodler and Celli, 2011), or (iii) bacteria escape into the host cell cytosol where they are linked with ubiquitinated proteins (Perrin et al., 2004; Knodler et al., 2010). Once in the cytosol, *S.* Typhimurium behavior depends on the type of cell in which they reside. In epithelial cell lines, escape into the cytosol leads to extensive bacterial proliferation, greater to that observed in SCV (Knodler et al., 2010), whereas in macrophages, the cytosol exhibits a bactericidal activity, leading to bacterial killing. In fibroblasts very limited proliferation of the pathogen has been described (Cano et al., 2001). *Salmonella* contributes to this limited proliferation, since bacterial overgrowth is observed upon inactivation of the PhoP/PhoQ two-component system which also controls expression of the T3SSs.

To date, it is still unknown whether these phenomena, observed mainly *in vitro*, reflect events occurring *in vivo*, and are of additional significance in terms of *S.* Typhimurium pathogenesis in animals. Whether these additional bacterial populations represent a successful host cell clearance mechanism or an *in vitro* artifact remains to be explored. A feature that distinguishes the *in vivo* behavior of intracellular bacteria is their limited capacity to proliferate inside host cells where only three to four individuals per infected cell have been observed (Sheppard et al., 2003). The most widely accepted model indicates that *S.* Typhimurium colonizes mouse organs by increasing the number of infection foci rather than increasing the number of intracellular bacteria per cell. Repetitive cycles of limited proliferation inside host cells followed by cell lysis and infection of neighboring cells may account for the increase in infection foci (Sheppard et al., 2003). It should be noted here that all these phenomena have not been described for plant cells, and the multiplication, localization strategies used by *Salmonella* in plant cells remain poorly understood.

All together, the different results presented in this chapter show that *Salmonella* could have multiple behaviors depending on the cells and the hosts considered. Moreover, we now know that *Salmonella* can enter cells through different mechanisms that could lead to different intracellular behavior. The impacts that these different intracellular behaviors have on host responses and stimulation of immune responses will undoubtedly be a new challenge in the future.

### HOST DEFENSES

Animals and plants differ quite extensively in the way they perceive and respond to invading organisms. However, for certain aspects they exhibit similarities. In both animal and plant kingdoms, when pathogens enter an organism, a rapid innate immune response is induced to impede the spreading of the pathogen. This response relies on both germline-encoded membrane-bound and intracellular receptors. In animals, this first line of defense is followed by an adaptive response in which genes encoding immune receptors and antibodies are subjected to somatic rearrangements, which allow the recognition of very specific epitopes of the pathogen. Specialized immune cells are activated and migrate upon production of soluble factors such as cytokines and chemokines. Finally, during adaptive response an immunological memory is developed which allows the production of an enhanced response in the event of a subsequent encounter with the same pathogen. In plants there are no specialized immune cells and their defense relies on the ability of the infected cell to recognize the pathogen and induce the adequate response. A zigzag model has been proposed to describe the general immune system in plants (Jones and Dangl, 2006). In this model conserved PAMP are recognized by membrane bound receptors triggering the PAMP triggered-immunity (PTI). Thereafter, successful pathogens inject effectors into the cell with the objective of interfering with PTI. These effectors are recognized by intracellular receptors, which launch the effector-triggered immunity (ETI), which eventually culminates in cell death known as HR. In addition, plants can develop two types of systemic resistance in which contact with pathogenic or non-pathogenic beneficial microorganisms induces resistance in distal parts of the plant. Systemic acquired resistance (SAR) is induced upon infection with pathogenic bacteria or fungi and protects the plant against a broad spectrum of pathogens. ISR, the second systemic resistance type, is the result of an interaction between soil rhizobacteria or mycorrhizal fungi and the host plant. The detection of *Salmonella* by animals as well as the induced defense response has been the object of abundant literature (Broz et al., 2012; Ruby et al., 2012; Wigley, 2013). In plants, due to the increasing number of outbreaks of disease associated with consumption of contaminated fruit or vegetables, more and more studies have recently focused on *Salmonella*–plant interactions and particularly on the host response (Fraiture and Brunner, 2014; Garcia and Hirt, 2014; Melotto et al., 2014).

#### Receptors of the innate immune response

***Extracellular receptors.*** In both animals and plants membrane-embedded receptors are in charge of detecting pathogens in the extracellular environment. They recognize conserved motifs within bacterial, viral, or fungal structures. In animals there are two main classes of these receptors: the C-type lectin receptors and the Toll-like receptors (TLR). TLR are by far the most studied because they play a key role in bacterial clearance. They are composed of three domains: a leucin-rich repeat (LRR) which is responsible for ligand fixation inducing homo- or hetero-dimerization of the receptor, a transmembrane, and an intracellular domain, which initiates the signaling cascade leading to activation of the host response. In plants there are two categories of extracellular receptors. The receptor-like kinases (RLK) encompass an extracellular domain, which may be an LRR, a lectin or a LysM domain, a transmembrane and an intracellular kinase domain. The receptor-like proteins (RLP) have an extracellular LRR and a transmembrane domain but lack the cytoplasmic part. A large difference in the number of extracellular receptors is observed between animals, where 13 TLRs have been described in the mouse, and plants where 200 RLPs and 600 RLKs genes have been identified in *Arabidopsis*.

In animals, TLR4 recognizes the LPS (Hoshino et al., 1999) in a complex multiprotein process involving at least four partners. The lipid A moiety of LPS is recognized by the LPS binding protein, then a ternary complex is formed with CD14 and finally LPS is delivered to the TLR4-MD2 complex (Park and Lee, 2013). TLR4 also recognizes the fibronectin (Okamura et al., 2001) and taxanes originating from plants and used as anti-tumor agents (Kawasaki et al., 2000). Very recently it has been shown that PrgI and SsaG, respectively, two structural proteins of *Salmonella* T3SS-1 and T3SS-2 needles, activate the innate response through TLR4 and also TLR2 (Jessen et al., 2014). In plants, despite the proven role of LPS in host defense (Shirron and Yaron, 2011), no associated receptor has so far been identified. In contrast to animals, both lipid A and the core oligosaccharide moieties of LPS are responsible for its immunostimulatory properties, the core oligosaccharide being involved in an early phase of the response and the lipid A in a later one (Silipo et al., 2005). Moreover Berger et al. (2011) have strongly suggested that the *O*-antigen from *Salmonella* may be considered as a PAMP in plants.

In both animals and plants, flagellin is an important PAMP recognized by extracellular receptors. A mutant strain of *Salmonella* deficient for the expression of flagellin has been shown to be able to colonize more efficiently *Medicago sativa* suggesting that *Salmonella* flagellin is recognized by the plant (Iniguez et al., 2005). *Arabidopsis* flagellin insensitive 2 (FLS2), a receptor of the RLK family, recognizes a 22-amino acid long peptide (flg22) from the N-terminus of the flagellin from different pathogens including *Salmonella* (Felix et al., 1999; Garcia et al., 2013; Meng et al., 2013). In animals, two conserved regions in the N and C terminal domains are recognized by TLR5. However, the flg22 motif is unable to activate innate immunity in animal cells (Donnelly and Steiner, 2002).

***Intracellular receptors.*** In order to enter the host cell and to survive, bacteria produce effector proteins which are translocated into the cytosol of the host cell through the T3SS-1 or the T3SS-2 apparatus. In animal and plant cells, cytosolic receptors have the ability to detect these effectors. In animals, receptors belonging to the TLR, the Nod-like receptor (NLR), the RIG-I like receptors (RLR), and the IFI200/HIN-200 (PYHIN) families are involved in detecting non-self determinants. In plants, the nucleotide-binding site-LRRs (NB-NLR) family encoded by the R-genes encompasses two subclasses of receptors the CC-NB-LRR and the TIR-NB-LRR. NLRs in animals and plants have a similar architecture with the LRR moiety conferring effector recognition specificity, a central domain responsible for receptor dimerization upon ligand fixation and an N-terminal domain which interacts with downstream signaling partners. To be fully functional, intracellular receptors are associated in multi-protein complexes. For example, infection of macrophages with *Salmonella* leads to the formation of a macromolecular complex encompassing ASC, NLRP3, NLRC4 caspase-1, caspase-8, and pro-IL-1β (Man et al., 2014). Interestingly, it has been shown that some members of vertebrate NLR and of plant NB-LRR receptors are both physically associated with HSP90 and SGT1 chaperones which are essential for the activation of innate immunity (Mayor et al., 2007). As for extracellular receptors, the number of NB-LRR in plants exceeds the number in animals with about 150 genes identified in *Arabidopsis* compared to around 20 in animals.

The question arises of how a limited number of receptors, especially in animals, can cope with the incredible diversity of non-self structures presented by pathogens. So far, direct interaction between NLR and their ligands has not been observed in animals. In plants there are at least two examples of direct recognition of effectors by R-protein. In *Arabidopsis*, a direct interaction has been shown between RRS1-R and the effector PopP2 (Deslandes et al., 2003) and in rice between the effector AvrPita and Pita (Jia et al., 2000). An interesting model, in which receptors detect modified self-proteins, has emerged from studies in plants (Dangl and Jones, 2001). In the guard model, the receptor is the guardian of a cellular protein (the guardee) it detects effector-induced modification of this protein and activates ETI. In this economy of means model, one receptor is able to detect modification of a host protein which may be the target of several pathogens. A given protein may be guarded by different receptors. The response of *Arabidopsis* to effectors from *Pseudomonas syringae* is one of the examples illustrating the guard model. In this model, the guardee protein RIN4 is targeted by different unrelated effectors (AvrRpm1, AvrRpt2, AvrB, or HopF2). AvrRpt2 is a protease, which cleaves RIN4. This cleavage is detected by the RPS2 receptor, which induces ETI. Both AvrB and AvrRpm1 phosphorylate RIN4, the receptor RPM1 recognizes the phosphorylated RIN4 protein and triggers ETI (Mackey et al., 2002, 2003; Wilton et al., 2010). The response of mice to the T3SS-1 SopE effector from *Salmonella* is evocative of the guard model in plants. When injected into the cytosol, SopE activates the small RhoGTPases Rac1 and Cdc42, this activation is detected by the receptor NOD1 and leads to the development of an inflammatory response (Keestra et al., 2013). In animals some *Salmonella* effectors are recognized by intracellular receptors. The T3SS-1 effector SipA activates NOD1/NOD2 (Keestra et al., 2011), while the T3SS-1 protein PrgJ and flagellin are recognized by the inflammasomes NLRC4-NAIP2 and NLRC4-NAIP5, respectively (Zhao et al., 2011; Halff et al., 2012). In *Nicotiana* benthamiana, the T3SS-2 effector SseF is probably recognized by a NB-NLR receptor (Ustun et al., 2012). In both animals and plants, recognition of effectors by their receptors launches signaling cascades which eventually lead to pathogen clearance.

#### Suppression of innate immune response by *Salmonella*

In animals, the interaction of innate immune receptors with their ligands may have two outcomes. The first is the activation of the key transcription factor NF-κB or of the MAPKs cascade, which ends with the transcriptional activation of numerous genes involved in inflammation, such as IL-6, iNOS, or TNFα. The second is the assembly of multiproteic scaffoldings, the inflammasomes, in which pro-caspase 1 is recruited and activated in an autocatalytic process leading to the maturation of pro-inflammatory cytokines like IL-1β or IL-18 and to a cell death known as pyroptosis. Plants possess a large family of MAPKs, some of which are involved in signaling cascades pivotal in PTI and ETI (Meng and Zhang, 2013). However important information on the intermediary signaling components which link receptor activation and the MAPK cascades is still missing. Induction of PTI and ETI induces overlapping responses including the production of ROS, antimicrobial compounds, signaling molecules like ethylene, salicylic acid, and jasmonic acid or enhanced expression of *pathogenesis*-*related* (*PR*) genes. In addition to these responses, HR is the usual outcome of ETI.

*Salmonella* can induce PTI in plants. For example, the MAPK cascade is activated in *Arabidopsis* inoculated with this bacterium (Schikora et al., 2008). On the other hand, inoculation of *Arabidopsis* with a *spiB* mutant leads to a higher number of bacteria in the roots compared to inoculation with wild-type *Salmonella*, raising the possibility that the T3SS-1 encodes proteins recognized by the plant immune system (Iniguez et al., 2005). Expression of the SseF in *Nicotiana benthamiana* induces the HR, a hallmark of ETI (Ustun et al., 2012). In tobacco, living *Salmonella* does not induce signs of defense response, while LPS from *Salmonella* or killed bacteria do, indicating that the bacterium is able to suppress the response, and this suppression is T3SS-1-dependent (Shirron and Yaron, 2011). *Arabidopsis* inoculated with a T3SS-1 mutant overexpressed genes associated with defense response when compared to inoculation with a wild-type *Salmonella* (Schikora et al., 2011).

However, *Salmonella* has implemented different strategies to overcome the defense response. In animals different *Salmonella* effectors may inhibit immune signaling pathways like NF-κB, the MAPK cascade or the transcription factor Syk through direct interaction with some signaling components (**Table 1**). *Salmonella* may also use some cellular intermediaries to inhibit the response. An unidentified protein from *Salmonella* activates the NLRP12 inflammasome, which in turn down-regulates NF-kB (Zaki et al., 2014). The bacterium may also target directly the receptor involved in its recognition. It has been shown that *Salmonella* down-regulates the expression of the intracellular receptor NLRC4 in B lymphocytes preventing the production of IL-1β and pyroptosis, allowing bacteria to stay hidden in lymphocytes (Perez-Lopez et al., 2013). Very recent data have uncovered different strategies used by *S*. Typhi to circumvent immune response. *S*. Typhimurium induces gastroenteritis and triggers inflammation with recruitment of neutropils to the intestine; in contrast, S. Typhi is associated with a systemic disease with little intestinal inflammation and few neutrophils. Typhi and Typhimurium serovars differ, with the former having a *via* locus. The *S.* Typhi *tviA* regulator gene indirectly downregulates the expression of HilA, a master regulator of the T3SS1, preventing recognition of SopE and activation of NK-κB (Winter et al., 2014). At the same time, chemotactism of the c5a component of the complement toward neutrophils is impaired by the Vi capsular antigen encoded in the *via* locus (Wangdi et al., 2014). In plants, there are few examples of modulation of immune response by *Salmonella*. The serovar Senftenberg, which differs in its canonical flg22 peptide, displays a reduced PTI when inoculated in *Arabidopsis* seedlings (Garcia et al., 2013) suggesting that some *Salmonella* strains may have evolved to escape recognition by FSL2. It has been shown that a mutant of *Salmonella* unable to assemble its T3SS1 apparatus is unable to suppress the expression of genes related to response to pathogens (Schikora et al., 2011) suggesting that some suppressor factors are injected in the cell by the T3SS1. Another study (Shirron and Yaron, 2011) has highlighted the suppressive activity of *Salmonella*: live bacteria do not produce oxidative burst in tobacco while heat killed bacteria or *Salmonella* LPS are able to do so. There is also an interesting example of cross-kingdom modulation of the immune response by the T3SS2 effector SspH2 (Bhavsar et al., 2013). In animal cells, this E3 ubiquitin ligase forms a ternary complex with STG1 which is a co-chaperone of the NLR NOD1; formation of this complex induces ubiquitination of NOD1, increases its activity, and stabilizes the SspH2 effector. STG1 which is highly conserved within eucaryotes, also interacts with SspH2 in plants enhancing their immune response.

**Table 1:** *Salmonella* effectors which inhibit immune signaling pathways.

Effector	Translocated by	Inhibit	Reference
AvrA	T3SS-1	NF-κB	Collier-Hyams et al. (2002)
		MAPK	Wu et al. (2012)
SseL	T3SS-2	NF-κB	Le Negrate et al. (2008)
SseK	T3SS-2	NF-κB	Li et al. (2013)
SspHl	T3SS-1	NF-κB	Haraga and Miller (2003)
SpvC	T3SS-1	MAPK	Mazurkiewicz et al. (2008)
SptP	T3SS-1	Syk	Choi et al. (2013)

## CONCLUSION

The ability of *Salmonella* to persist outside its hosts is a critical trait that enables this pathogen to occasionally contaminate fresh produce and therefore cause food-borne disease outbreaks. The ability of the human enteric pathogens to exploit plants as alternative hosts has emerged as an important area of research in the last decade. It has become apparent that *Salmonella* not only passively survives on or within plants but also actively infects them. However, contrary to *Salmonella* with animals or animal cells, these interactions have not been well characterized. Some common features have been identified such as the use of the T3SS or the way animals and plants detect this pathogen. Future studies are required to investigate whether mechanisms employed by *Salmonella* to infect animals and plants are similar. These studies should lead to improved understanding of the evolution of host specificity and will have important impacts on risk assessment and food protection.

## Conflict of Interest Statement

The Guest Associate Editor, Nicola Holden, declares that, despite having collaborated with author, Adam Schikora, the review process was handled objectively and no conflict of interest exists. The authors declare that the research was conducted in the absence of any commercial or financial relationships that could be construed as a potential conflict of interest.
